# The risks and bottlenecks to automation in employment in Argentina. New impacts on the occupational structure in selected economic sectors

**DOI:** 10.3389/fsoc.2026.1755111

**Published:** 2026-03-19

**Authors:** Eduardo Chávez Molina, José Rodríguez de la Fuente

**Affiliations:** 1 Instituto de Investigaciones Gino Germani, Universidad de Buenos Aires, Buenos Aires, Argentina; 2 Departamento de Sociología, Universidad Nacional de Mar del Plata, Mar del Plata, Argentina; 3 Instituto de Investigaciones Gino Germani, Universidad de Buenos Aires, Consejo Nacional de Investigaciones Científicas y Técnicas, Buenos Aires, Argentina

**Keywords:** artificial intelligence, automation, economic sectors, employment, occupational structure

## Abstract

This study investigates the automation risk in employment in four urban areas of Argentina, analysing the impacts of technological changes and the digital economy on the occupational structure. A survey (*N* = 426) was conducted using the Respondent Driven Sampling (RDS) technique. The analysis covered sectors with different levels of technological integration—high (e.g., software), medium (e.g., food), and low (e.g., textiles). An automation risk index was constructed based on job skills and tasks. The results indicate that professionals, scientists, managers, and technicians exhibit a lower risk of automation, while elementary and industrial occupations face a higher risk. Social and creative intelligence were identified as ‘bottlenecks’ in the face of automation, an aspect that we have emphasised in this analysis. The software and pharmaceutical sectors are more protected, unlike the textile and hotel industries. In addition, the use of Artificial Intelligence (AI) is more prevalent in lower-risk occupations, mainly for complex and skilled tasks, complementing human work. In conclusion, the study emphasises the need to understand these changes in order to comprehend and predict the future of the labor market. Skills involving emotional and creative intelligence offer robust protection against automation, although emerging generative AI could begin to impact tasks previously considered secure.

## 1 Introduction

Technological changes, and with them robotisation and the advancement of automated machines, together with Artificial Intelligence (AI), are creating a new landscape, not only in terms of the cultural changes that this implies, but also in terms of business strategies in the corporate world, and mainly in terms of new working conditions for employees. Although the process of technological change is heterogeneous, segmented and multifaceted, the penetration of technological innovations in the business world is evident in the digital transformation and the scope of the digital economy, which are taking on different speeds and scales according to operating and capital conditions.

Changes in the business world, especially when it comes to transforming business models, have an impact on workers’ conditions, occupations, tasks, qualifications, expectations, as well as on recruitment, retraining, or exclusion processes in new procedures that are implemented. In recent years, part of the discussion on changes in labor markets has focused on which occupational segments were most exposed to advances in computing and robotisation. Thus, the initial focus was on the impact that advances in computing had on routine tasks, both analytical and interactive as well as symbolic, and on the polarisation that this process caused in jobs and incomes (Autor et al., 2003; Acemoglu and Autor, 2011). Subsequently, attention turned to the effects that mobile robotics and machine learning could have on jobs involving non-routine cognitive tasks (Frey and Osborne, 2017) and, more recently, the focus has shifted to the impacts of generative AI (GenAI) on occupations requiring higher levels of education and training (Eloundou et al., 2023; Gmyrek et al., 2023).

In this context, the article seeks to identify and characterize the extent and distribution of automation-related risks in the labour market in four urban areas in Argentina in 2024. The results focus on a variety of industries with different levels of technological incorporation (high, medium, and low). As a secondary objective, the paper contributes to the discussion on the development of tools aimed at refining and strengthening instruments for analysing automation-related risks in employment. Given the methodological limitations involved in extrapolating automation indices for use in national labour surveys, we rely on original primary data collected through a survey that follows the structure of the Programme for the International Assessment of Adult Competencies (PIAAC). Based on this information, we propose the construction of an automation index derived from the survey data.

Unlike existing measures based on occupational classifications, the proposed index adopts a task-based and individual-level approach, which allows for capturing within-occupation heterogeneity in exposure to automation risks. The index seeks to illustrate the analytical value of original task-level data in the Argentine context and to complement existing occupation-based indicators by focusing on broader bottlenecks to automation identified in the literature.

The study does not aim to provide nationally representative estimates. However, the selection of large urban areas responds to specific analytical criteria: they concentrate a high degree of productive heterogeneity and account for approximately 62% of the country’s employed population (EPH-INDEC, second quarter of 2025).

Moreover, most quantitative studies on labour automation conducted in Argentina have relied on household surveys and on the adaptation of automation indices developed for other national contexts. These strategies entail well-known limitations, particularly issues of compatibility with the occupational classification system currently in use in the country. With the exception of Zukerfeld (2025), few studies have employed primary data sources specifically designed to capture the content of work tasks. In this regard, the present study seeks to contribute to filling this gap through the use of original data and a task-based approach.

The article is organized into four sections. The first section reviews the main theoretical and methodological approaches that have been developed in the recent literature on automation-related risks in employment. Here, special emphasis is placed on recent advances in research in Latin America. Next, we describe the survey and questionnaire used to address labor automation in Argentina. The results section is divided into three sections: a preliminary exploratory analysis of the main tasks considered for the study of automation risk; the development of the index; and a descriptive analysis of automation risks. Finally, the discussion summarises the main results of the research.

## 2 Recent approaches to the risk of automation

### 2.1 Task- and occupation-based approaches

Concerns about the advance of automation in the workplace have been a recurring issue in the social sciences. Industrial revolutions have enabled ever-increasing production capacity and scope, generating, at each stage, concern about the possible replacement or transformation of the tasks that workers have performed (Espíndola and Suarez, 2023, p. 55), which has been described in various studies as automation anxiety (Autor, 2015). However, the emergence of the so-called fourth industrial revolution, driven by STARA technologies (Smart Technology, Artificial Intelligence, Robotics and Algorithms) that can automate cognitive and non-routine tasks, has raised further questions about the magnitude of the impact on work (Espíndola and Suarez, 2023).

We can say that, during the first two decades of the 21st century, at least two major approaches to studying the risk of automation in the labor market were established. The first of these was the task-based approach developed by Autor et al. (2003), Acemoglu and Autor (2011), Autor and Dorn (2013), and Acemoglu and Restrepo (2020). This analytical model, which emerged from the study of technological advances between 1960 and 2000, posits that computers were able to replace routine tasks (cognitive and manual) that could be performed by following programmed rules. In contrast, non-routine tasks, which require problem solving, complex communication, flexibility, creativity, or expert judgement, could be complemented by technology. This approach predicted a polarisation of the labor market, with the growth of high- and low-skilled jobs at the expense of intermediate ones, a phenomenon observed in the United States between 1980 and 2000. The particularity of this approach is that it understands work tasks that must be observed and not occupations as a homogeneous whole.

The second approach, which we can call “occupation-based,” is proposed by Frey and Osborne (2017). This approach (FO) updates the previous model by arguing that, due to advances in the fields of machine learning, mobile robotics and the use of big data, a greater number of non-routine cognitive tasks are susceptible to automation. In this regard, the authors identify “bottlenecks” that limit automation: perception and manipulation, creative intelligence, and social intelligence. According to the authors, the technology of the 2010s could not yet match the breadth and depth of human perception, creativity, persuasion, negotiation, and care for people (Frey and Osborne, 2017, p. 262). This approach predicted a higher risk of automation, including low-skilled jobs that were previously considered less exposed: 47% of US employment was at high risk of future automation.

Although the FO model identified the bottlenecks to automation based on tasks, their methodology led to the automation score being assigned directly to a set of 702 occupations that could be coded using the Occupational Information Network (O*NET) database. This type of implementation led to a series of studies pointing to an overestimation of the risk of automation by Frey and Osborne, assuming that it is entire occupations that are automated and not specific tasks. Thus, starting from the same bottlenecks, but from a task-based approach, other research finds that the risk of automation was reduced to 9–14% of employment for OECD countries (Arntz et al., 2016; Nedelkoska and Quintini, 2018). The greatest risk of automation would fall on low-skilled workers and those jobs that require a basic level of education. By contrast, the least automated occupations would almost all require vocational training and/or higher education.

### 2.2 The impact of generative AI: new measures of automation

With the advances made in the field of Artificial Intelligence, mainly in Machine Learning, Deep Learning and Natural Language Processing, studies on the risks of automation in the labor market have once again focused their attention on future impacts. A series of investigations (Webb, 2019; Felten et al., 2021; Lassébie and Quintini, 2022) began to suggest that the development of technologies for the recognition and generation of images, sounds, and text can affect, to a greater extent, high-skilled occupations with high educational requirements. Mainly, Lassébie and Quintini (2022) find that the most exposed are those that involve reading comprehension tasks, deductive and inductive reasoning skills, and programming skills, although they question the idea that occupations involving these skills are completely automated.

However, with the massive irruption of GenAI –through GPTs and other Large Language Models (LLMs)–the future of work and automation anxiety once again took centre stage. The novelty of this type of AI lies in its ability to create new content (images, text, sounds or code) from patterns learned in large training datasets, in a relatively user-friendly way. In this context, researchers from OpenAI (Eloundou et al., 2023), the company that developed ChatGPT, analysed the impact of this technology on the labor market. According to their estimates, 80% of the U.S. labor market may see at least 10% of their tasks affected by LLMs and 19% may have 50% or more of their tasks affected. To do this, they employed a novel methodology using human annotators and GPT-4 as a classifier on occupational data in the U.S. economy (O*NET). The main results showed that programming and writing skills were the most susceptible to being replaced by LLMs. In line with this approach, Felten et al. (2023), find that occupations with higher incomes, higher educational levels and greater creative skills are more exposed to automation by GenAI, i.e., those workers who were more protected against previous technological advances.

Following a methodology similar to that of Eloundou et al. (2023), the ILO recently developed a global index of occupational exposure to AI (Gmyrek et al., 2023, 2025). However, unlike the other approaches that used O*NET as a data source, this proposal is based on the International Standard Classification of Occupations (ISCO-08), with the definitions of its 436 occupations and 3,123 tasks. Using GPT-4 they have constructed an index that scores occupations from 0 (minimum exposure) to 1 (maximum exposure). The greatest impact of GenAI is among administrative support workers, followed by technicians and professionals (i.e., occupations related to “knowledge”). The authors point out that the risk of replacement of an occupation is also linked to the range and variability of tasks it presents. In this way, occupations with highly diversified tasks can take greater advantage of complementation with GenAI. On the other hand, occupations that are very homogeneous in tasks that can be replaced by the GenAI would have a higher risk of being replaced (for example: Data Entry Clerks, Accounting and Bookkeeping Clerks, Statistical, Finance and Insurance Clerks, General Office Clerks, etc.).

Despite this, some authors argue that the future impact of GenAI on work will depend on several factors. A key determinant is the level of trust that humans place in this technology and how humans adapt their habits. The cost of technology, the preferences of workers and employers, and incentives influence the adoption of these tools (Eloundou et al., 2023, p. 22). Frey and Osborne (2023) have also been sceptical about the disappearance of the bottlenecks to automation that they had identified. According to them, the importance of tasks involving human interaction (managerial, professional, customer-oriented occupations) will be particularly valuable skills. Likewise, the importance of human trust will only grow with the implementation of LLMs. About creative work, GenAI would have a significant impact, but with limitations in generating new narratives or conceptual leaps. The training data used by LLMs would be exhausted and they would begin to train based on their own products. Thus, the content generated by GenAI would be of low quality and biased.

### 2.3 Approaches implemented in Latin America

In Latin America, analyses have been carried out on the risk of labor automation, applying the FO approach (Aboal and Zunino, 2017; Weller et al., 2019) and the task-based approach (Egana-delSol et al., 2022). However, the same studies warned of the theoretical and methodological limitations that the application of this index could bring to the region. When applying automation risk estimation models, it is necessary to consider the lag in the implementation of new technologies, especially in sectors with low economic productivity. Furthermore, the lack of appropriate surveys to measure the impact of automation on work tasks forced the use of household surveys by applying averages to aggregate occupational categories, generating biases in the results (Chávez Molina et al., 2024). Some exceptions are those studies that have used the PIAAC surveys and the Skills for Employment and Productivity survey (STEP–World Bank) (Egana-delSol et al., 2022; Martinez, 2023).

One of the few comparative studies carried out in the region (Martinez, 2023), applying the FO approach but combined with PIAAC data, calculated the risk of automation for 14 countries, reaching conclusions similar to those of Frey and Osborne (2017). The risk increased in: people with a medium level of education, men, sectors of medium and high productivity and the middle classes. More recently, Egana-delSol and Bravo-Ortega (2025) have analysed the risk of AI automation in five Latin American countries, concluding that it has a differential impact on those occupations that require greater educational training and skills.

In Argentina, specific studies have also been carried out extrapolating various indices to microdata from the Permanent Household Survey (Gasparini et al., 2020; Chávez Molina, 2023; Chávez Molina and de la Rodríguez Fuente, 2023) or from the task-based approach (Maurizio and Monsalvo, 2020). The estimates, using the FO approach, reach values somewhat higher than those reported in the original study for the American case. Conversely, there is still little research that has addressed the impact of GenAI on the national labor market (Ministerio de Trabajo, Empleo y Seguridad Social, 2023; Zukerfeld et al., 2023).

## 3 Data

### 3.1 Sample

The article discusses the impact of automation based on the results of a primary source. The target population consists of salaried workers employed in firms with different levels of technological incorporation, operating in selected industries and urban labour markets in four urban areas of Argentina (Autonomous City of Buenos Aires -CABA-, districts of Greater Buenos Aires^1^ -GBA-, Córdoba and Mar del Plata).

The study universe was delimited in three scales (Schumpeter, 2021). Firstly, companies with high technological incorporation, those organisations that are characterized by intensively integrating advanced technologies into their production processes, in the development of their products or in the provision of their services. These organisations not only transform their own sectors, but also have a significant impact on the economy and society by boosting productivity and generating new markets (Castells, 2011; OECD, 2015). The sample includes the branches of computer science, metalworking and pharmaceuticals.

Secondly, companies with medium technological incorporation were identified. They refer to those organisations that integrate technology into their processes and products, but not as intensively or advanced as high-onboarding companies. They tend to operate in sectors such as traditional manufacturing, livestock, specialized services, or construction, where technology plays an important role but is not the main driver of their competitiveness (Soete and Freeman, 2012). The analysis covers the food industry and the hospitality industry (hotels and restaurants).

Finally, companies with low technological development, i.e., those organisations that use basic or traditional technologies in their production processes and in the production of their products or services. Unlike companies with high or medium technological incorporation, these organisations do not invest significantly in research and development (R + D) or adopt cutting-edge technologies. However, they play a crucial role in the economy, especially in developing countries, as they generate employment and contribute to social and economic stability (OECD, 2015). The sample considered the textile industry (clothing and footwear) and furniture.

Given the absence of a reliable sampling frame and the segmented structure of labour networks across industries and occupations, the survey was conducted using “Respondent Driven Sampling” (RDS) (Heckathorn, 1997) designed to collect data in a systematic and structured way. This technique was particularly useful for accessing hard-to-reach populations, such as informal workers or professionals in specific job niches. For example, in a previous pilot study, RDS identified and recruited workers in the platform economy (such as digital riders or freelancers)^2^ who would otherwise have been underrepresented in traditional samples. This survey stood out for its ability to capture detailed information about participants’ perceptions and behaviors, which proved critical for subsequent statistical analysis. In addition, RDS made it easier to identify patterns and trends in the data, such as the relationship between job satisfaction and flexibility, and the measurement of automation “bottlenecks”.

In this type of sampling, implementation begins with the purposive selection of a small set of initial participants, or “seeds,” whose diversity is intended to facilitate penetration into different segments of the social network, without requiring them to be representative. The expansion mechanism relies on a dual incentive system: a primary incentive for participation and, crucially, a secondary incentive that rewards the successful recruitment of peers, typically limited to three traceable coupons per participant in order to control the disproportionate influence of highly connected individuals. This process generates chains of peer referral, the structure of which is recorded confidentially. In parallel, each respondent is administered an instrument that measures their “network degree”—that is, the size of their personal network within the target population—as well as key characteristics of their contacts.

Recruitment started from an initial set of seeds selected purposively to maximise heterogeneity in terms of industry, occupation, gender, and educational level, and proceeded through successive recruitment waves following the RDS protocol.

Information from the Permanent Household Survey of the National Institute of Statistics and Census (EPH-INDEC) on the labour composition of the selected industries was used as a reference to guide the proportional distribution of recruitment across urban areas. Recruitment outcomes varied across cities in terms of chain length and seed productivity: while some initial seeds did not generate further recruitment, others produced one or more recruitment waves. Table 1 summarizes the number of initial seeds by city and their recruitment outcomes.

**Table 1:** Initial seeds and recruitment outcomes by urban area.

Urban area	Seeds with no recruitment	Seeds with 1 wave	Seeds with ≥2 waves	Total seeds
CABA	12	10	16	38
GBA	21	14	16	52
Córdoba	26	14	3	43
Mar del Plata	4	11	0	15

As part of the RDS protocol, respondents were asked to report the approximate number of people they personally knew who met the study’s eligibility criteria, in order to measure individual network size. However, RDS-based weights were not applied in the analyses. Instead, the sample was adjusted using demographic weights by urban area, based on the relative population size of each urban area. This decision reflects the exploratory nature of the study and its focus on identifying task-level patterns and relationships among variables rather than producing population-level estimates.

Therefore, the sample should be understood as non-probabilistic. Despite the use of RDS, certain groups (particularly highly educated professionals and technicians) are overrepresented, while informal and low-skilled workers remain underrepresented. For this reason, the results should be interpreted as sample-specific patterns that illuminate task-level dynamics and potential automation bottlenecks within the studied context, rather than as representative estimates of the Argentine labour market as a whole.

Data collection was carried out through *SurveyCTO* software between the months of May and July 2024. The final sample consists of 426 observations. Table 2 summarises the main demographic characteristics of the sample across industries.

**Table 2:** Sample demographic characteristics.

Characteristic	Overall *N* = 426	Software *N* = 90	Metalworking *N* = 100	Pharmaceutical *N* = 30	Food *N* = 103	Hospitality *N* = 25	Textile *N* = 58	Furniture *N* = 20
Sex								
Man	282 (66.2%)	69 (76.7%)	96 (96.0%)	14 (46.7%)	50 (48.5%)	13 (52.0%)	22 (37.9%)	18 (90.0%)
Woman	144 (33.8%)	21 (23.3%)	4 (4.0%)	16 (53.3%)	53 (51.5%)	12 (48.0%)	36 (62.1%)	2 (10.0%)
Age group								
19–29	128 (30.0%)	40 (44.4%)	26 (26.0%)	2 (6.7%)	38 (36.9%)	11 (44.0%)	6 (10.3%)	5 (25.0%)
30–39	119 (27.9%)	40 (44.4%)	25 (25.0%)	3 (10.0%)	28 (27.2%)	6 (24.0%)	15 (25.9%)	2 (10.0%)
40 and older	179 (42.0%)	10 (11.1%)	49 (49.0%)	25 (83.3%)	37 (35.9%)	8 (32.0%)	37 (63.8%)	13 (65.0%)
Place of residence								
Autonomous City of Buenos Aires	120 (28.2%)	54 (60.0%)	14 (14.0%)	16 (53.3%)	13 (12.6%)	3 (12.0%)	18 (31.0%)	2 (10.0%)
Greater Buenos Aires	128 (30.0%)	11 (12.2%)	35 (35.0%)	11 (36.7%)	37 (35.9%)	5 (20.0%)	20 (34.5%)	9 (45.0%)
Córdoba	91 (21.4%)	10 (11.1%)	42 (42.0%)	0 (0.0%)	25 (24.3%)	2 (8.0%)	10 (17.2%)	2 (10.0%)
Mar del Plata	87 (20.4%)	15 (16.7%)	9 (9.0%)	3 (10.0%)	28 (27.2%)	15 (60.0%)	10 (17.2%)	7 (35.0%)
Highest educational level attained								
Up to completed secondary	304 (71.4%)	59 (65.6%)	81 (81.0%)	12 (40.0%)	72 (69.9%)	17 (68.0%)	45 (77.6%)	18 (90.0%)
Completed higher education	122 (28.6%)	31 (34.4%)	19 (19.0%)	18 (60.0%)	31 (30.1%)	8 (32.0%)	13 (22.4%)	2 (10.0%)

Source: own calculations based on the 2024 automation risk survey.

### 3.2 Questionnaire

For the preparation of the questionnaire, the guidelines of the OECD PIAAC survey were followed. This survey fundamentally evaluates reading comprehension, mathematical skills and problem solving in digital environments of the population over 16 years of age. As far as our research is concerned, the questionnaire covers a number of interests in:

- reading and math activities
- the use of ICTs at work and in daily life
- skills such as collaborating with others and organizing one’s own time
- autonomy at work

Although only two rounds of the survey were conducted, in 2011–2018 and 2022–2023, in Argentina it has never been carried out.

On the other hand, the main variables from the O*NET, a widely used reference source at the international level, were also identified and evaluated. The O*NET database established itself as a key resource due to its comprehensiveness and methodological rigor, providing standardized information on a wide range of occupations in the U.S. labor market. The primary purpose of this database is to provide detailed and structured information on a wide variety of occupations, making it an invaluable resource for labor analysis. The data collected include aspects such as the skills required, the knowledge required, the tasks performed, the activities carried out and the working conditions associated with different jobs (Peterson et al., 2001).

The design of the instrument considered the following dimensions: sociodemographic information, education and training, respondent’s work, tasks and activities at work (interaction, learning, organization, physical work), cognitive skills (reading, writing, numbers, problems), use of technology and use of generative AI. The full questionnaire is available as Supplementary material to this article.

Following the proposals of Arntz et al. (2016) and Espíndola and Suarez (2023), the dimensions that represent the “bottlenecks” to automation are operationalized in the FO approach (2017) (Table 3). However, the dimension related to the perception and manipulation of objects from the original proposal was not incorporated. Recent studies (Lassébie and Quintini, 2022) indicate that tasks involving cramped workspaces and awkward positions, finger dexterity, and manual dexterity exhibit a higher degree of automatability. These indicators were nevertheless included in the questionnaire, but they did not allow for a clear discrimination of tasks within the surveyed population.

**Table 3:** Dimensions and indicators for the construction of the automation index.

Dimension	Skills
Social intelligence	Cooperate with other workers
	Share work-related information
	Teach
	Advise
	Plan your own activities
	Plan activities for others
	Organize your own time
	Influence
	Negotiate
Creative intelligence	Solve simple problems
	Solve complex problems
	Calculate fractions, decimals, or percentages
	Use advanced math and statistics
	Reading articles in newspapers, professional journals
	Reading diagrams, maps, diagrams
	Article Writing
	Reports Writing
	Use programming languages

Source: own elaboration on Arntz et al. (2016) and Espíndola and Suarez (2023).

## 4 Results

### 4.1 Exploratory analysis

In this section we describe some of the most important dimensions and variables for the study of automation that have been collected in the survey. First, we observe how the main labor variables are distributed. Most of the surveys were carried out among workers in the food industry (24.2%), metalworking workers (23.5%) and software (21.1%). The sample presents a dominance of scientific and intellectual professionals with 36.2%, followed by artisans and workers in related trades with 22.5%. This is mainly due to the activities analyzed, where the tasks that are carried out in areas of complex labor development and high-skilled tasks are highlighted. To a lesser extent, workers in unskilled elementary activities, and directors/managers.

#### 4.1.1 Social intelligence

Regarding the indicators that account for the “bottlenecks” to automation, we will first review the tasks and work activities linked to social intelligence, that is, those that involve interaction and organization.

**Figure 1:**
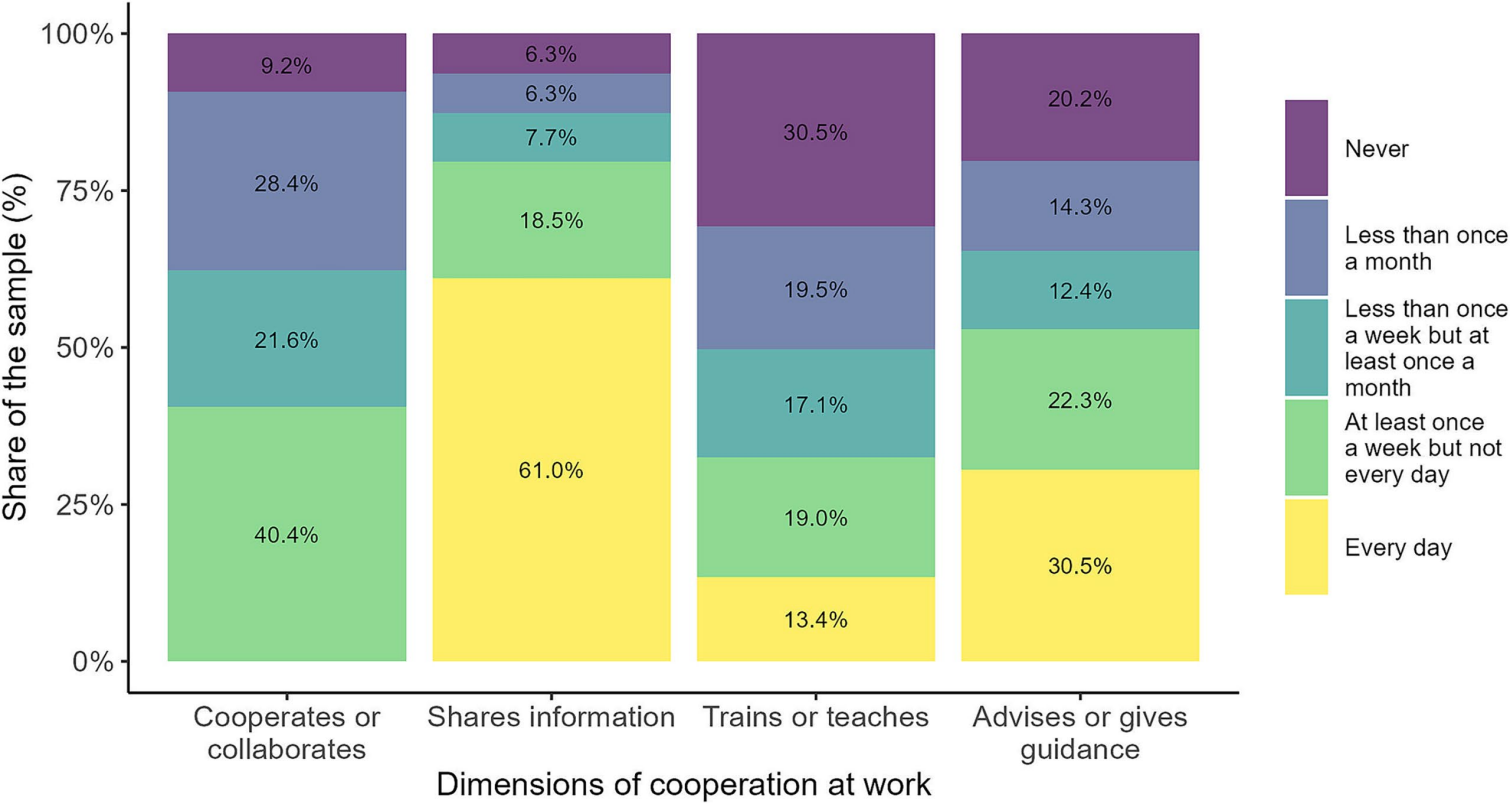
Dimensions of cooperation at work. The figure illustrates workers' engagement in cooperative activities at work across four key dimensions. Each bar represents the distribution of responses regarding the frequency with which individuals report cooperating or collaborating, sharing information, training or teaching others, and advising or providing guidance. Source: own calculations based on the 2024 automation risk survey.

The survey collected various indicators related to cooperation among workers (Figure 1). In this regard, “shares information” and “advises or gives guidance” are activities that are performed with notable daily frequency (61 and 30.5%, respectively). By contrast, “trains or teaches” are less widespread tasks. In general, cooperating or collaborating with others appears to be an activity that no respondent reported performing daily, while four out of ten reported doing it at least once a week.

**Figure 2:**
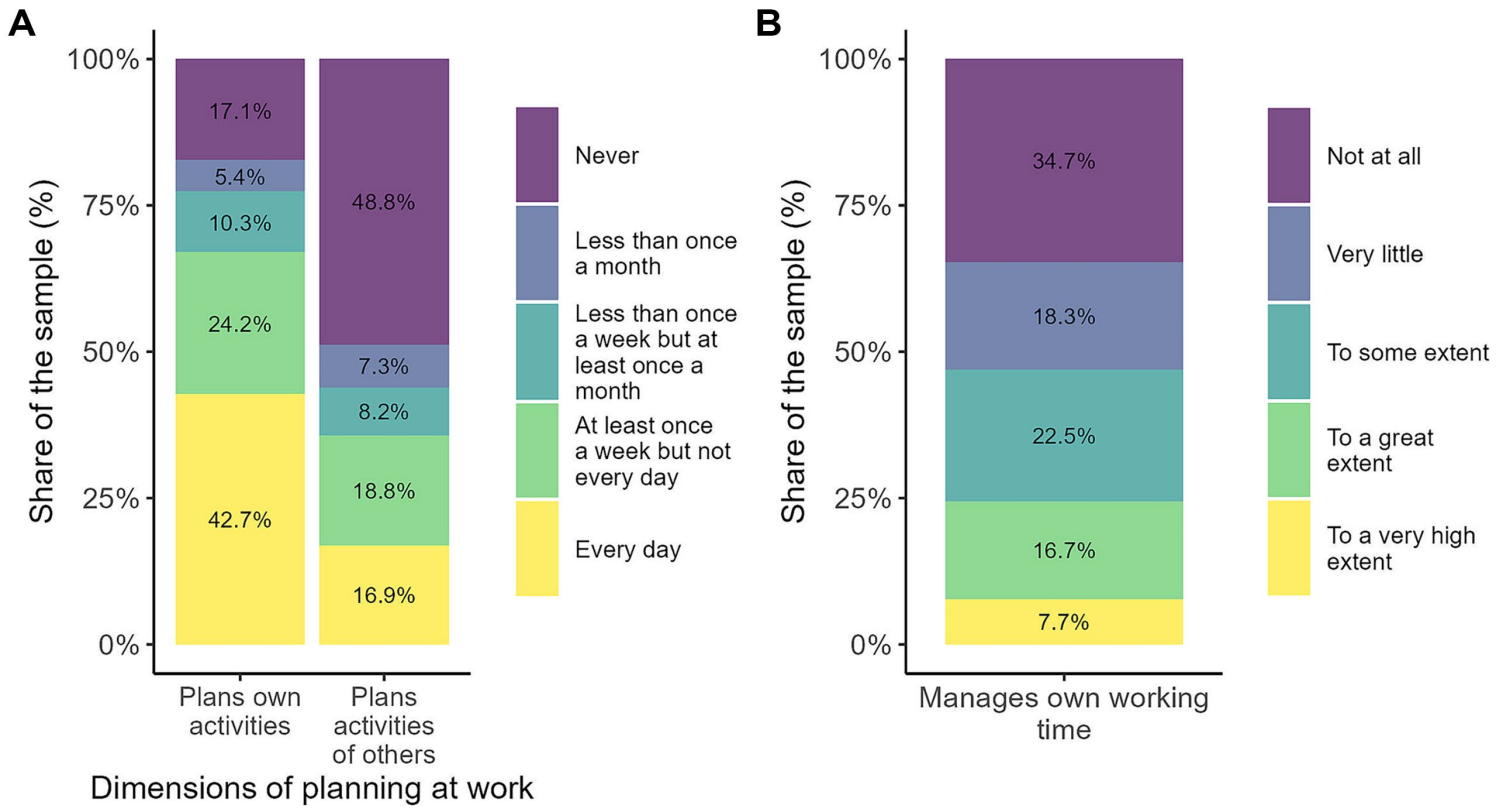
Dimensions of planning at work. The figure presents the distribution of workers' planning activities at work across three main dimensions. In **(A)** each bar illustrates the frequency with which respondents report planning their own activities and planning the activities of others, while **(B)** shows the extent to which workers report being able to manage their own working time. Source: own calculations based on the 2024 automation risk survey.

Figure 2 shows that planning one’s own activities is a common and frequent practice among workers. A substantial share report doing it every day, indicating a strong tendency toward self-organisation and continuous adjustment to changing work demands. Planning at least once a week is also widespread, suggesting that regular structuring of tasks remains important even when not done daily. In contrast, planning the activities of others is far less common: nearly half of respondents report never doing so, reflecting that most positions do not involve supervisory or managerial responsibilities, which tend to concentrate this type of planning.

However, planning one’s own work activities does not necessarily imply greater autonomy over one’s own time. Only 24% of respondents reported being able to manage their own time to a great or very great extent.

**Figure 3:**
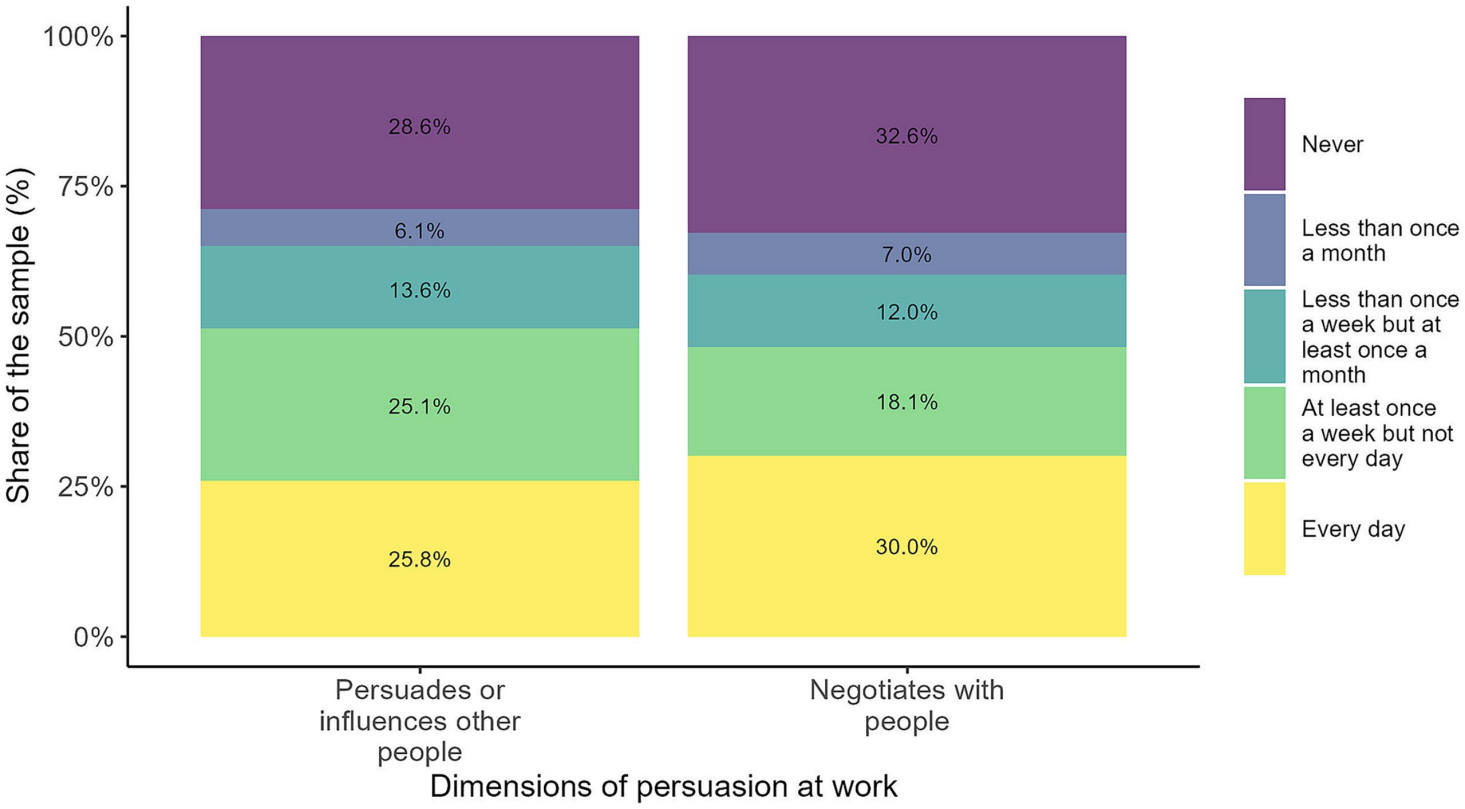
Dimensions of persuasion at work. The figure presents the distribution of workers' persuasion-related activities at work across two dimensions: persuading or influencing other people and negotiating with people. Each stacked bar shows the share of respondents reporting how often they perform each activity, ranging from "every day" to "never". Source: own calculations based on the 2024 automation risk survey.

Persuasion-related activities (Figure 3) constitute an important component of social intelligence at work, although they are unevenly distributed across occupations. Around a quarter of workers report persuading or influencing others on a daily basis, highlighting the relevance of this skill in roles involving leadership, coordination, or client interaction. An additional share performs these activities at least weekly, indicating that persuasion is a regular part of many work routines. Negotiation shows a similar pattern: while some workers engage in it frequently, a substantial proportion never does so, reflecting its concentration in specific positions such as management or sales.

#### 4.1.2 Creative intelligence

**Figure 4:**
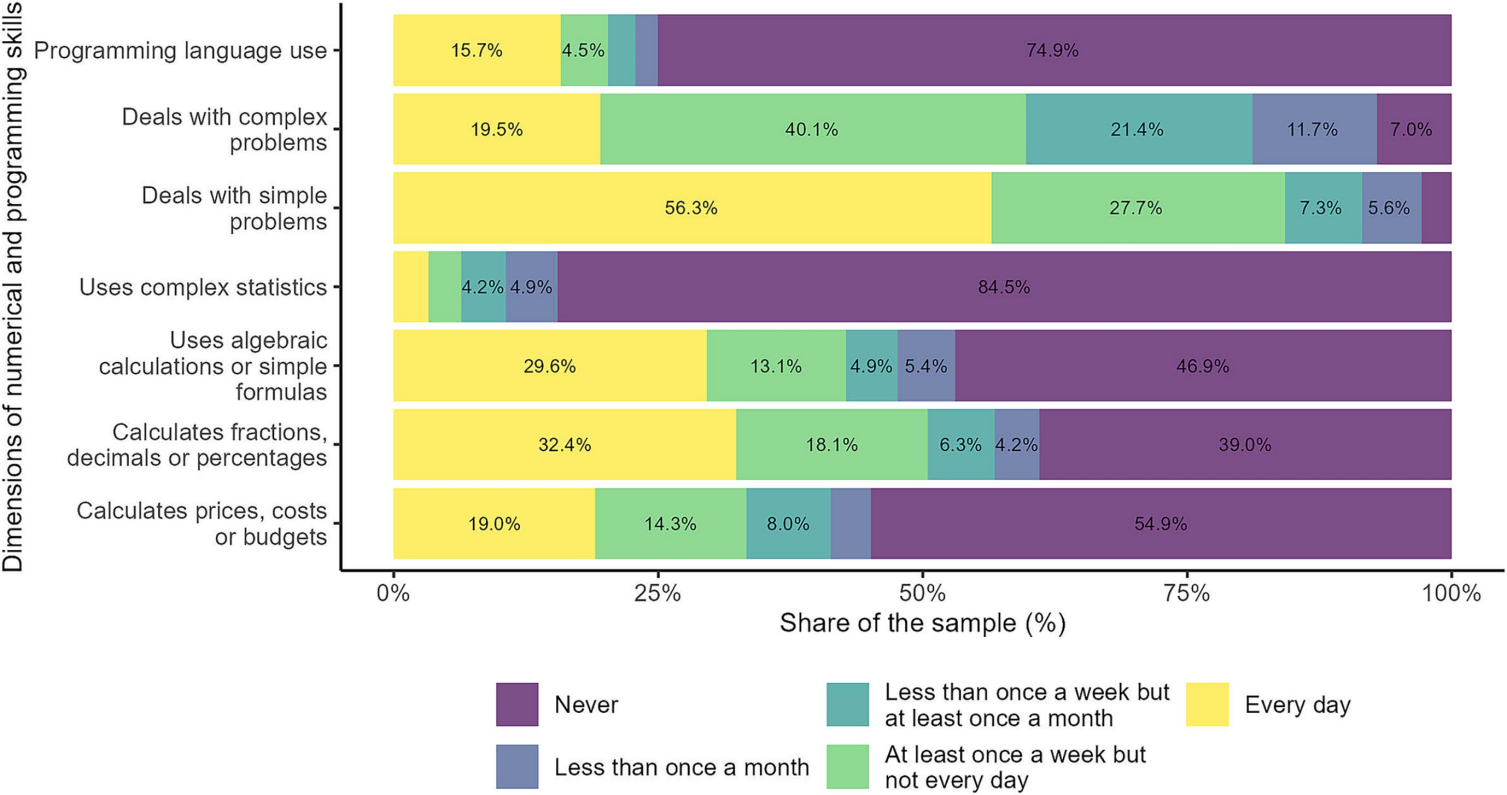
Numerical and programming skills. The figure illustrates workers' self-reported use of numerical and programming skills across seven different tasks. Each bar represents the distribution of responses regarding the frequency with which individuals engage in activities such as calculating prices, costs, or budgets; working with fractions, decimals, or percentages; applying algebraic calculations or simple formulas; utilizing complex statistics; solving simple or complex problems; and programming language use. Source: own calculations based on the 2024 automation risk survey.

Figure 4 shows that problem-solving activities and calculations vary in frequency depending on their complexity. Simple problems and basic calculations, such as fractions and percentages, are more common and are performed more frequently. On the other hand, complex problems and algebraic calculations are less frequent, suggesting that they are needed in more specific or specialized contexts. In general, basic math skills and financial management are integral to daily life for a large portion of respondents, while more advanced skills (complex statistics and use programming language) are used by a smaller group. This reflects how practical needs influence the frequency of these activities.

**Figure 5:**
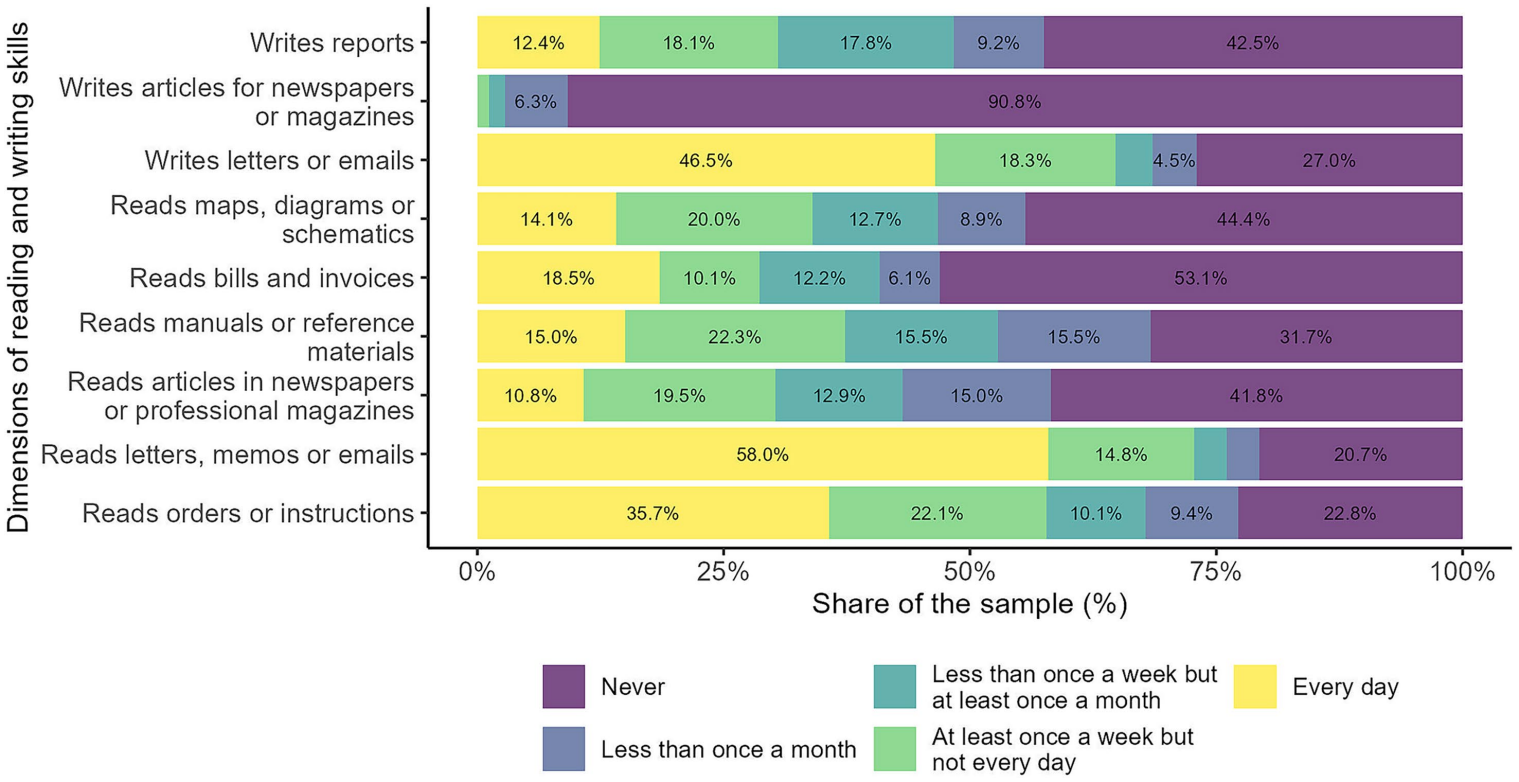
Reading and writing skills. The figure illustrates workers' self-reported use of reading and writing skills across nine different tasks. Each bar represents the distribution of responses regarding the frequency with which individuals engage in activities such as reading orders or instructions; reading letters, memos, or emails; reading articles in newspapers or professional magazines; reading manuals or reference materials; reading bills and invoices; reading maps, diagrams, or schematics; writing letters or emails; writing articles for newspapers or magazines; and writing reports. Source: own calculations based on the 2024 automation risk survey.

The frequency of use of reading and writing skills is also stratified according to the level of complexity (see Figure 5). While almost the majority of respondents do not write articles, the vast majority frequently read or write emails. Writing reports and reading maps, diagrams, and articles is more evenly distributed.

#### 4.1.3 Use of AI

**Figure 6:**
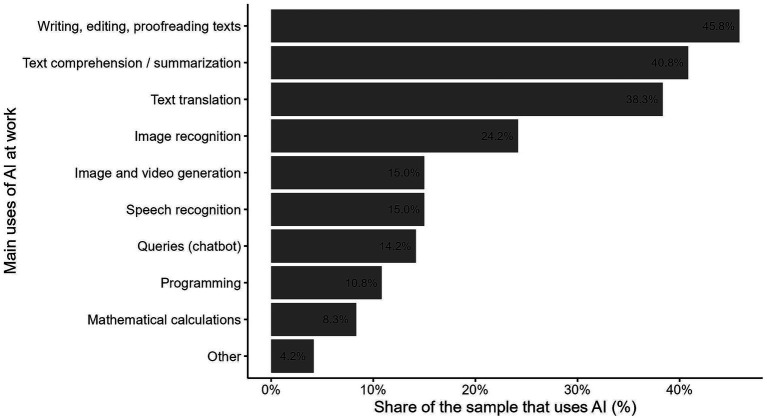
Share of the sample that uses AI. The figure shows the percentage distribution of Al-using surveyed workers by the main uses of Al at work. The horizontal axis reports the share of Al users associated with each type of use, while the different Al use categories are listed on the vertical axis. Source: own calculations based on the 2024 automation risk survey.

Finally, the survey asks about the use of Artificial Intelligence (AI) to facilitate work tasks. In this context, 28% of the sample stated that they made some use of it. Figure 6 shows the main tasks performed by the people surveyed under the use of this technology.

Writing, editing and proofreading of texts occupies the highest percentage (46%), closely followed by the comprehension and synthesis of texts (41%). Text translation also has a significant representation (36%). Image recognition (24%) and image and video generation are less prevalent (15%) but still play an important role. In a similar range, there is audio recognition (15%) and queries through *chatbots* (14%). Programming and math calculations have lower percentages compared to the other tasks.

In summary, it can be observed that, although a small proportion of the surveyed population uses AI-related tools, their use is mainly based on tasks widely found in various segments of the occupational structure, linked to the use of text. This is associated with the generalisation that Large Language Models (LLMs) have begun to experience (Gmyrek et al., 2023).

### 4.2 Automation index construction

After the exploratory analysis of the dimensions and variables that allow us to observe how certain skills and work tasks are distributed among the people in the sample, we advance in the elaboration of a synthetic measure that works as an index on the risk of labor automation. To do this, we will use multiple correspondence analysis (MCA) (Le Roux and Rouanet, 2010), which allows us to reduce the complexity of the association between all the variables to a single factor that condenses the largest explained variance.^3^ This technique has been widely used to construct indices (Filmer and Pritchett, 2001; McKenzie, 2005; OCDE et al., 2025).

A total of 18 variables, presented in Table 3, were included in the MCA. The first factor accounts for 55% of the total inertia, while the second explains 18% (Table 4). Frequency categories were coded as follows: N (“Never”), LM (“Less than once a month”), LW (“Less than once a week, but at least once a month”), AW (“At least once a week, but not every day”), and ED (“Every day”). In addition, levels of agreement or intensity were coded using the following categories: N (“Not at all”), VL (“Very little”), SE (“To some extent”), GE (“To a great extent”), and VHE (“To a very high extent”). The MCA was estimated including all observed categories as active, with missing values automatically excluded by the procedure. No frequency-based exclusion was applied *a priori*.

**Table 4:** Modified inertia and cumulative inertia of the MCA.

Axis	Inertia (%)	Cumulative inertia (%)
1	54.99	54.99
2	18.18	73.17
3	6.74	79.91
4	3.86	83.77
5	3.05	86.82
6	2.42	89.24
7	1.79	91.02
8	1.57	92.59
9	1.18	93.77
10	1.15	94.92

Inertia values correspond to modified eigenvalues obtained using the Benzécri correction, as implemented in the GDAtools package. Source: own calculations based on the 2024 automation risk survey.

**Figure 7:**
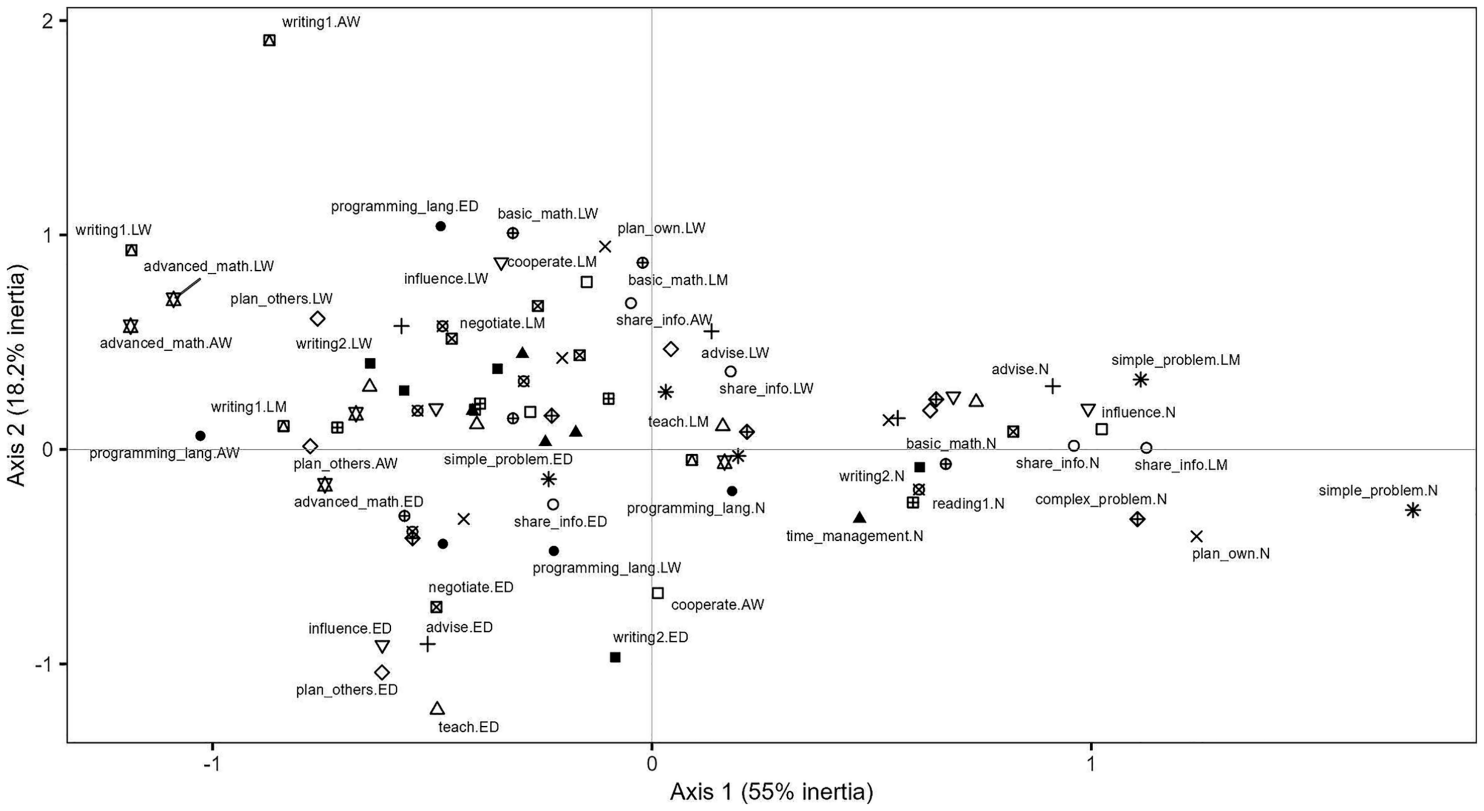
Multiple correspondence analysis (task and abilities). The figure presents the factorial map from a MCA of work-task indicators. Points represent the modalities of each variable; proximity between modalities indicates association (frequent co-occurrence), while distance from the origin reflects their contribution to the overall variation. Axes 1 and 2 account for the largest share of total inertia (55% and 18.2%, respectively). Source: own calculations based on the 2024 automation risk survey.

Figure 7 displays the cloud of active variable categories resulting from the multiple correspondence analysis. Axis 1, which accounts for the largest share of explained inertia (55%), primarily differentiates workers according to the frequency with which they perform the tasks considered. The left-hand side of the axis is associated with higher frequencies of task performance (e.g., at least once a week or every day), whereas the right-hand side concentrates categories corresponding to the non-performance of these tasks (“never”). Tasks contributing most strongly to this dimension include influencing, advising, planning one’s own and others’ work, negotiating, the use of basic mathematics, teaching, and reading.

Axis 2 (18.2% of inertia) introduces a further distinction within the group of workers who perform these tasks, separating those who carry them out on a daily basis (ED) from those for whom task performance is less frequent (e.g., at least once a week or less). This dimension thus captures differences in the intensity of task execution rather than simple presence or absence.

Taken together, the configuration of the factorial plane suggests that workers located on the left-hand side of the graph—characterized by a higher and more intensive engagement in cognitive, social, and organizational tasks—are relatively more sheltered from automation-related risks, whereas those positioned on the right-hand side exhibit task profiles that are comparatively more exposed, from a task-content perspective (Frey and Osborne, 2017).

**Figure 8:**
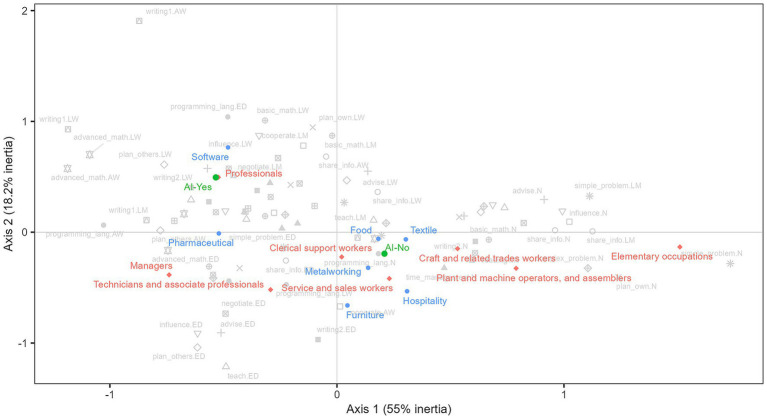
Multiple correspondence analysis. Tasks and skills. Supplementary variables (occupations, industry, and use of AI). The figure presents the factorial map from a MCA of work-task indicators (in grey), together with the projection of supplementary variables (in color). Supplementary modalities-such as occupational groups (red), sectors/industries (blue), and Al use (green)-are included for interpretative purposes only and do not contribute to the construction of the axes. Source: own calculations based on the 2024 automation risk survey.

Figure 8, projects occupational categories (ISCO-08), industries, and the use of artificial intelligence as supplementary variables onto the factorial plane (Le Roux and Rouanet, 2010). The resulting configuration suggest that occupations and industries differ systematically in the frequency and intensity with which particular types of tasks are performed. In this sense, professional, scientific, managerial, and technical occupations are located closer to categories associated with a higher frequency of cognitive, analytical, and organizational tasks, which are comparatively less exposed to automation-related risks. Conversely, elementary occupations (e.g., cleaning services, street vending) and operator occupations in industrial activities are more strongly associated with task profiles characterized by lower frequencies of such tasks and a greater concentration of routinized activities, which are relatively more exposed to automation.

A similar pattern emerges at the sectoral level. Industries such as software development and pharmaceuticals are positioned near categories corresponding to frequent engagement in complex, non-routine tasks, indicating lower exposure to automation risks.

On the other hand, the use of artificial intelligence appears to be more intensive in professional, managerial and technical occupations, where tasks related to mathematical calculation, programming, and writing are performed more frequently. In line with recent contributions (Eloundou et al., 2023; Felten et al., 2023; Gmyrek et al., 2025), these findings suggest that AI adoption in such occupations is more likely to be associated with task complementation or augmentation rather than full substitution. However, given the rapid and ongoing diffusion of AI technologies in the world of work, the real-time impact of AI remains difficult to assess.

From a task-based perspective, the higher prevalence of AI use among these workers is consistent with the idea that AI is more readily adopted in activities that have been identified as “bottlenecks” in theoretical frameworks (tasks that are cognitively demanding, non-routine, and difficult to automate fully). Nonetheless, these results should be interpreted with caution, as the evidence on AI use is exploratory in nature and based on a relatively small subgroup of respondents reporting AI use (28% of the sample). Moreover, the implications of generative AI for skilled work remain heterogeneous and context-dependent, varying across sectors and occupational roles.

The automation risk index is obtained from the factorial scores of the first factor of the MCA. These scores are rescaled to a 0–1 interval using a min–max normalization, according to the following formula:


$$AI_{i}=\frac{F_{i1}-\min(F_{1})}{\max(F_{1})-\min(F_{1})}$$

Where $F_{i1}$ denotes the factorial score of individual i on the first MCA factor. Under this transformation, values closer to 0 indicate lower exposure to automation-related risks, while values closer to 1 indicate higher exposure. In the resulting distribution, the index has a mean of 0.39 and a standard deviation of 0.20.^4^

It is important to clarify that the automation risk index developed in this study differs conceptually and methodologically from more recent proposals that focus specifically on exposure to AI, such as the index developed by the ILO (Gmyrek et al., 2025). In particular, this study builds on the framework originally proposed by Frey and Osborne (2017, 2023), which emphasizes key bottlenecks to automation related to machine learning and mobile robotics rather than focusing exclusively on AI applications. By collecting primary data explicitly designed to capture task content, the proposed index allows for within-occupation heterogeneity in task performance, an aspect that cannot be observed when automation risks are imputed solely on the basis of occupational titles.

Although the index is constructed using a non-probabilistic sample and is therefore not intended to produce nationally representative estimates, its contribution lies in demonstrating the feasibility and analytical value of a task-based approach using original survey data in a context where such information is largely unavailable. In the Argentine case, to the best of our knowledge,^5^ this is the first study to construct an automation risk indicator based on primary task-level data, rather than on the adaptation of indices developed for other national contexts. As such, the index should be understood as a complementary tool that enriches existing occupation-based measures by providing finer-grained evidence on how automation risks emerge from the tasks actually performed by workers.

### 4.3 Distribution of automation risks

Once the index has been constructed, we examine how the risk of labor automation is distributed across different segments of the sample. To this end, a set of demographic, educational, and labor-related factors is considered in order to assess how distinct groups within the sample are positioned with respect to automation risk. Importantly, all measures are derived from individual-level data, as the index is constructed on the basis of the tasks performed by each worker. The selected factors therefore serve to characterize individuals’ positions within broader labor market contexts, rather than representing aggregate or firm-level outcomes. To facilitate interpretation, the figures display the overall median and the 25th and 75th percentiles of the index as reference points.

**Figure 9:**
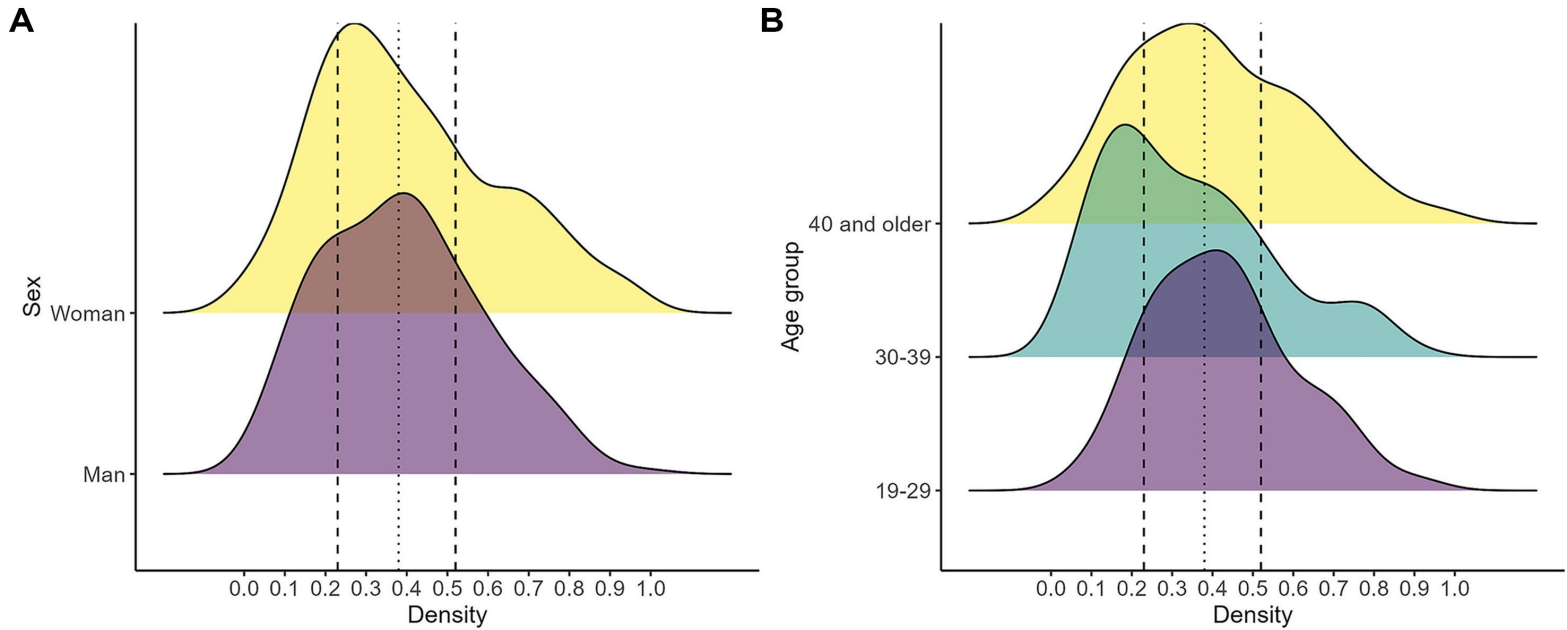
Automation risk by sex **(A)** and age **(B)**. The figure displays kernel density estimates of the distribution of the automation index across demographic groups. Panel **(A)** compares the distribution by sex, while Panel **(B)** compares it across age groups. The dotted vertical line marks the median of the overall distribution, and the dashed vertical lines indicate the 25th and 75th percentiles. Source: own calculations based on the 2024 automation risk survey.

According to the constructed index, higher levels of exposure to automation risk are observed among younger workers (19–29 years old) and men. As shown in Figure 9, the distribution for younger individuals is shifted to the right of the overall median, with a greater concentration of observations above the 75th percentile of the sample distribution. In contrast, workers aged 40 and over tend to be located closer to, or below, the median level of automation risk.

The greater exposure of young workers to automation risk is consistent with previous regional studies (Aboal and Zunino, 2017; Weller et al., 2019) and is largely related to their more precarious labor market insertion, particularly during the early stages of their working trajectories, when employment is more frequently concentrated in routine and low-autonomy tasks.

With respect to sex, the literature does not offer a univocal conclusion regarding gender differences in automation exposure (Acemoglu and Restrepo, 2020; Egana-delSol et al., 2022). However, regional evidence reported by ECLAC (Espíndola and Suarez, 2023) points in the same direction as the results presented here, with men displaying a distribution more concentrated at higher levels of automation risk. A plausible explanation is that women are relatively more likely to be employed in jobs that rely more intensively on social, communicative, and creative skills, which are currently less susceptible to automation.

**Figure 10:**
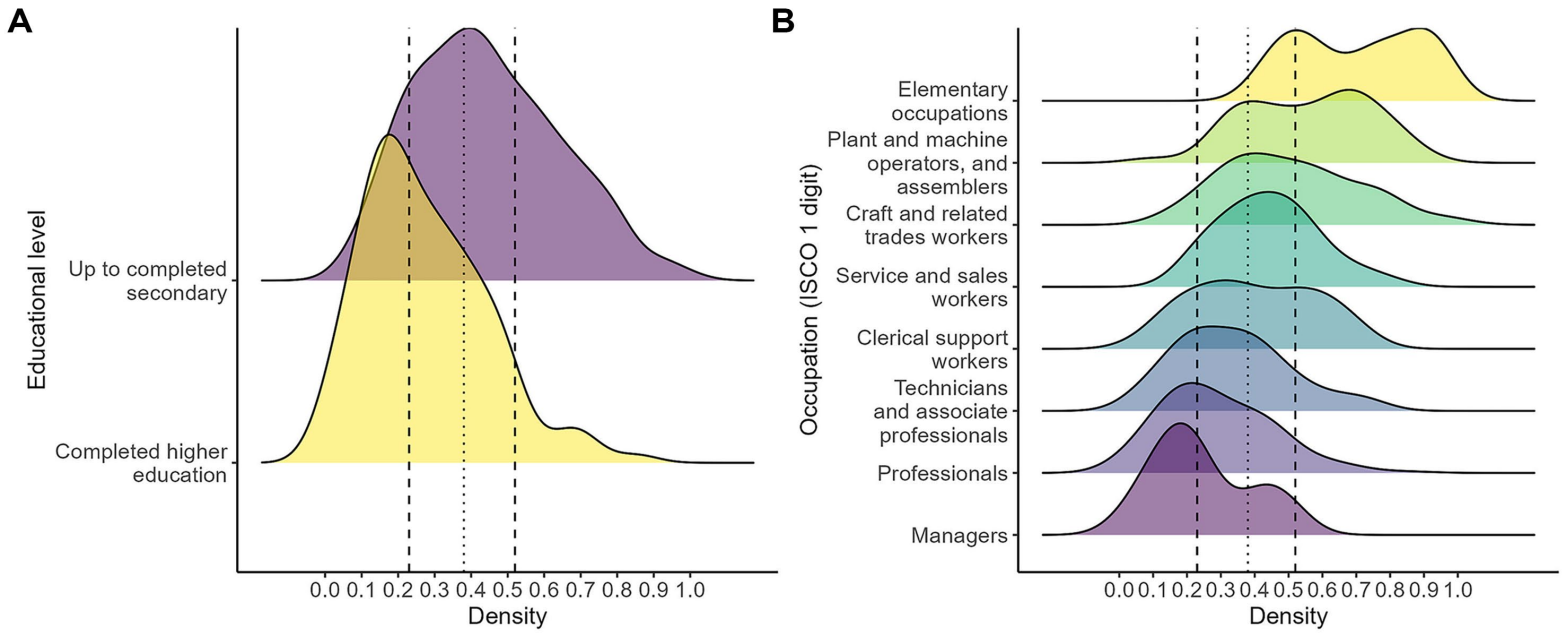
Automation risk by educational level **(A)** and occupation **(B)**. The figure displays kernel density estimates of the distribution of the automation index across labor market groups. Panel **(A)** compares the distribution by educational level, while Panel **(B)** compares it across occupational groups (ISCO 1 digit). The fitted vertical line marks the median of the overall distribution, and the dashed vertical lines indicate the 25th and 75th percentiles: own calculations based on the 2024 automation risk survey.

Regarding educational attainment and occupational position (Figure 10), the distributions indicate higher levels of automation risk among individuals with lower levels of formal education and those employed in occupations characterized by lower degrees of specialization and task complexity. As shown in Panel A, workers with up to completed higher education exhibit a distribution shifted to the right of the overall median, with a larger share of observations located above the 75th percentile of the sample distribution, compared to those who have completed higher education.

Panel B reveals a clear occupational gradient: elementary occupations, plant and machine operators, and craft-related trades tend to concentrate at higher values of the automation index, while professionals, technicians, and managers are more strongly represented at lower levels of risk. These patterns are consistent with the task-based nature of the index, as occupations involving routine, standardized, or manual tasks tend to be more exposed to automation than those requiring abstract reasoning, problem-solving, or interpersonal skills.

For Argentina, similar educational and occupational gradients in exposure to automation risk have been documented by Gasparini et al. (2020) and Aboal and Zunino (2017), lending further support to the plausibility of the patterns observed in this sample.

**Figure 11:**
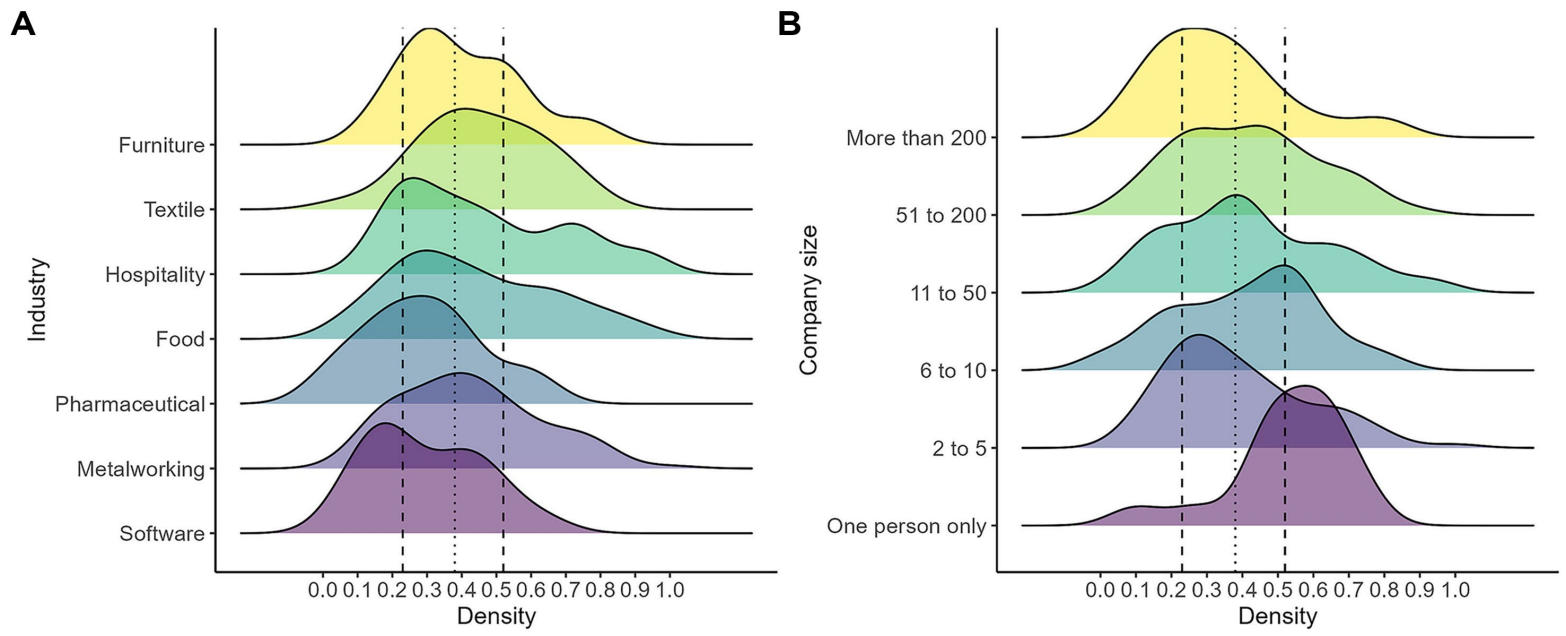
Automation risk by industry **(A)** and size of the establishment **(B)**. The figure displays kernel density estimates of the distribution of the automation index across labor market groups. Panel **(A)** compares the distribution by industry, while Panel **(B)** compares it across company size. The fitted vertical line marks the median of the overall distribution, and the dashed vertical lines indicate the 25th and 75th percentiles: own calculations based on the 2024 automation risk survey.

When looking at the probabilities of automation by industry (Figure 11), no very marked trends are identified: workers in industries such as furniture, textile, hospitality, and food display distributions that are shifted toward higher values of the index, with a substantial share of observations located above the sample median and within the upper interquartile range. Regarding the firm size, there are no clear patterns either, but there is a greater risk in cases of self-employment and in small establishments (6 to 10 employed).

**Figure 12:**
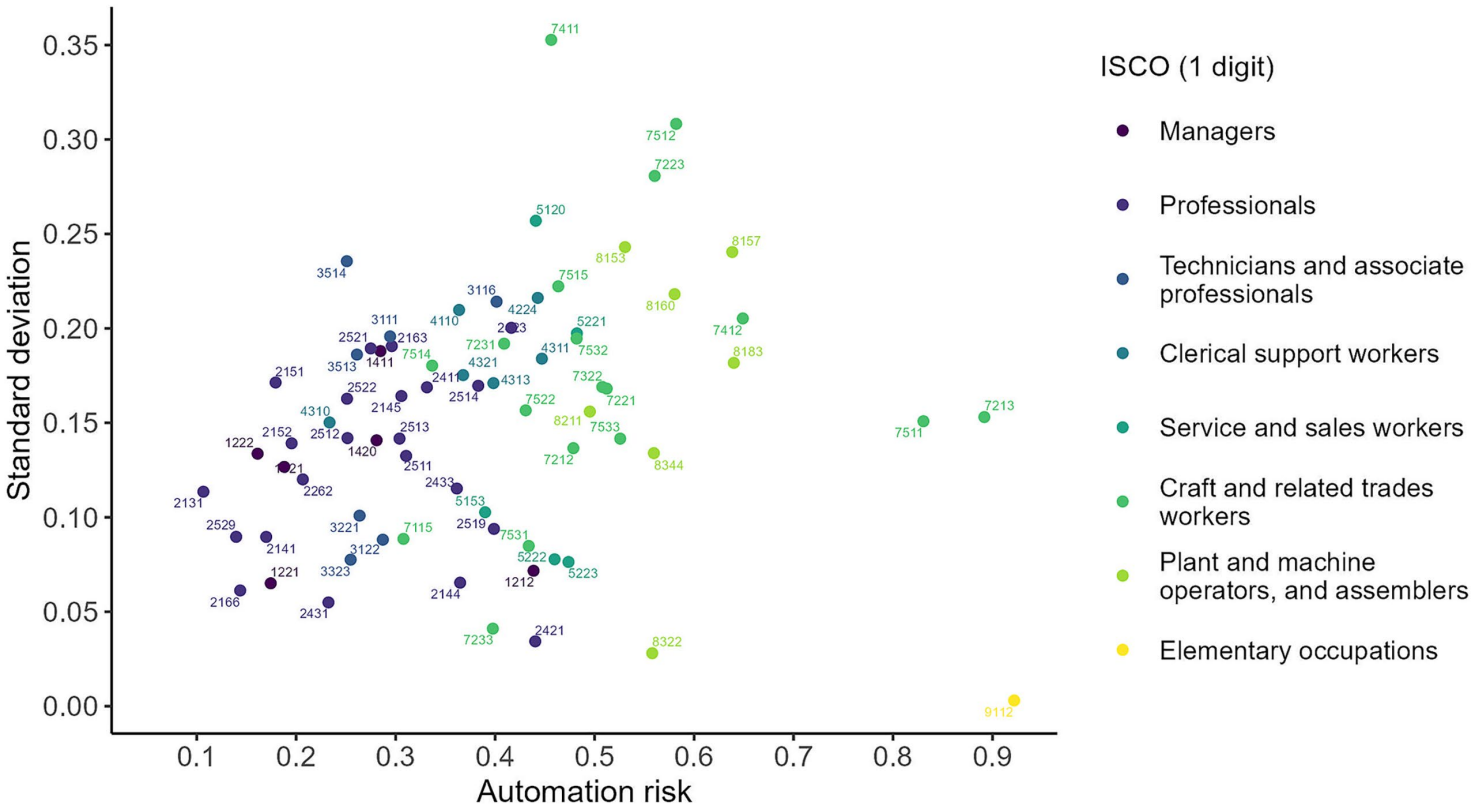
Distribution of occupations according to average and standard deviation of automation risk. The figure plots occupational-level automation risk against within-occupation dispersion in the automation index. Each point corresponds to a 4-digit ISCO occupation, colored by the 1-digit ISCO major group. The horizontal axis reports the estimated automation risk for each occupation, while the vertical axis shows the standard deviation of the index within that occupation. Source: own calculations based on the 2024 automation risk survey.

Finally, following the approach proposed by Gmyrek et al. (2023, p. 25), considering occupations as a set of tasks with different levels of exposure to automation, we focus on both the average and the standard deviation of the index. Thus, for each occupation coded in 4 digits of ISCO-08, we assess not only its risk of automation but also its degree of heterogeneity, at the level of the tasks performed. In Figure 12 we can identify occupations with a high risk of automation, but also with a low standard deviation, i.e., occupations strongly exposed to replacement due to the high exposure of all their tasks. In this case, occupations located in the right side include: Cleaners and assistants in offices, hotels and other establishments (9112), sheet metal workers and cauldrons (7213), and butchers and fishmongers (7511).

On the other hand, occupations located in the lower left sector have a low average risk and a great homogeneity in the tasks performed: biologists (2131), directors (1,221, 1,222), industrial engineers (2141), and database professionals (2529). On average, in the upper central end, there are occupations with a medium risk of automation and with a high heterogeneity in the exposure of the tasks that make them up. It is in these cases that it is most understood that there can be a process of complementation (*augmentation*) between automation and human work: cooks (5120), site electricians (7411) and sewing machine operators (8153).

This approach to the research problem highlights that some occupations are characterized by relatively homogeneous task profiles across workers, whereas others exhibit substantial within-occupation heterogeneity in the tasks performed. This perspective reinforces the idea that automation does not replace occupations per se (Frey and Osborne, 2017), but rather specific tasks within occupations (Autor et al., 2003; Arntz et al., 2016).

## 5 Discussion

In the current context of constant economic and technological change, it is crucial to have studies that reflect contemporary realities. This study provides recent evidence derived from original primary data collected in selected urban labor markets and industries in Argentina, with the aim of contributing to ongoing analytical and policy-oriented debates on labor automation.

The analysis focuses on cross-sectional differences in working conditions and task composition and their association with automation risk across different segments of the surveyed sample. To this end, industries with different levels of technological incorporation were analyzed, identifying three scales: high, medium and low technological incorporation. This approach allowed us to capture a comprehensive view of how technology impacts the world of work and how companies adapt to these changes.

First, Building on the contributions of Frey and Osborne (2017, 2023) and the task-based framework of PIAAC (Arntz et al., 2016), the paper proposes a data collection instrument specifically designed to operationalize key bottlenecks to automation. Second, although we start from the authors’ proposal, we construct an automation risk index from using individual-level task data, allowing us to capture within-occupation heterogeneity that cannot be observed through occupation-based indices alone. In this sense, the proposed index complements existing measures, such as those based on occupational classifications, by illustrating the analytical value of primary, task-level data in contexts where such information is scarce. As noted throughout the article, the proposed index does not aim to capture the effects of the recent and rapid diffusion of generative artificial intelligence on occupations (Gmyrek et al., 2025). However, by exploring the relationship between AI use and task content, the analysis shows that the use of these technologies is more prevalent among surveyed workers who perform tasks requiring higher levels of social and creative intelligence, as well as non-routine cognitive processes.

The results found show that the “bottlenecks” to automation identified in literature (Arntz et al., 2016; Frey and Osborne, 2017; Espíndola and Suarez, 2023), mainly in tasks that involve the use of social and creative intelligence, are strongly associated with the socio-occupational structure observed in the sample. Professional, scientific, managerial, and technical occupations tend to be more closely linked to tasks with lower automation risk, while elementary occupations and industrial operator roles are more strongly associated with tasks and skills at higher risk. At the same time, a set of intermediate occupations—especially in commerce and skilled manual work—exhibits substantial heterogeneity in task composition, combining activities that may be automated, complemented, or remain reliant on human labor.

With respect to industry, the software and pharmaceutical sectors appear, within the cases analyzed, to be among the least exposed to automation risk. However, workers who are inserted in these sectors are the ones who, to a great extent, are making the most intensive use of the different modalities of the GenAI. This resource, however, is still limited to tasks related to writing, interpretation, text translation and programming, that is, the use of Large Language Models. Although it is still a recent phenomenon, several authors (Acemoglu and Restrepo, 2020; Hatzius et al., 2023) are beginning to assess the extent to which this new wave of automation driven by GenAI may begin to increase the risk of task replacement in occupations that until recently were in a situation of greater protection.

Finally, while the findings should be interpreted in light of the study’s non-probabilistic design, sensitivity analyses indicate that the main patterns observed are robust to alternative specifications of the automation index. Future research would benefit from extending this approach to probabilistic samples, additional sectors, and longitudinal designs, in order to deepen the empirical understanding of automation dynamics and their implications for labor markets.

## Data Availability

The raw data supporting the conclusions of this article will be made available by the authors, without undue reservation.

## Footnotes

1 The Autonomous City of Buenos Aires and its surrounding districts (districts of Greater Buenos Aires) form a single urban agglomeration, despite being administratively separate jurisdictions.

2 Survey of delivery workers by platforms of the Social and Cultural Observatory for Sustainable Development (OSCDS) is an institutional space dependent on the Secretariat of Science and Technology of the National University of Córdoba.

3 Data processing was conducted using R (R Core Team, 2025) and the GDAtools package (Robette, 2024).

4 As a stringent robustness check, we recomputed the automation risk index after excluding the nine task variables with the highest contribution to the first MCA dimension (corresponding to half of the 18 active variables included in the analysis). Although these variables play a central role in defining the dimension, the resulting index remains strongly correlated with the baseline specification (r = 0.76). This indicates that the index captures a broader and coherent task structure rather than being driven by a small subset of highly influential variables.

5 An exception is the Occupational Survey (EncO) carried out by the Argentine Observatory of Occupations (OOA) under the Secretariat of Labour, Employment and Social Security during 2023–2024 in registered private-sector firms located in major urban agglomerations. However, the project was not completed and the data are not accessible.
