# Supplementary material for: The risks and bottlenecks to automation in employment in Argentina. New impacts on the occupational structure in selected economic sectors
Source: Front Sociol. 2026 Mar 19;11:1755111. doi: 10.3389/fsoc.2026.1755111 (PMC13043417; doi:10.3389/fsoc.2026.1755111)
Supplement: Supplementary file 1 [file Table_1.docx]

| **Sample information (for the interviewer)** |
| --- |
| Branch of activity:  ☐ Software  ☐ Metalworking  ☐ Pharmaceutical  ☐ Feeding  ☐ Hospitality  ☐ Textile  ☐ Furniture |
| City of activity:  ☐ CABA  ☐ GBA Metropolitan Area  ☐ Cordoba  ☐ Mar del Plata |
| **Sociodemographic information** |
| 1. Name: |
| 1. Month and year of birth: |
| 1. How old are you? |
| 1. Gender:   ☐Women  ☐Male  ☐Non-binary  ☐Other: |
| 1. Are you currently...   ☐ united?  ☐ married?  ☐ separated or divorced?  ☐ widowed?  ☐ single? |
| 1. Do you have children?   ☐ Yes  ☐ No |
| 1. Children grid  \| Son Name 1 \| Child age 1 \| \| --- \| --- \| \| Child Name 2 \| Child age 2 \| \| Son Name 3 \| Age 3 \| \| Son Name 4 \| Child age 4 \| \| Son Name 5 \| Child age 5 \| \| Child's name 6 \| Child age 6 \| \| Son Name 7 \| Age 7 \| \| Son Name 8 \| Child age 8 \| \| Son Name 9 \| Child age 9 \| \| Son Name 10 \| Age 10 \| |
| 1. What is your current health coverage? (indicate the main one or the one you use the most)   ☐ Social work  ☐ Prepaid  ☐ Public hospital or health centers  ☐ Another: |
| 1. Where was he born?   ☐ In this locality  ☐ In another town in this province  ☐ In another province (specify)  ☐ In a bordering country (specify: Brazil, Bolivia, Chile, Paraguay, Uruguay)  ☐ In another country (specify) |
| 1. **Education and training** |
| 1. Do you attend or attend any educational establishment? (college, school, university)?   ☐ Yes, attend  ☐ He does not attend, but he attended  ☐ He never attended |
| 1. That establishment is/was...   ☐ Public  ☐ Private  ☐ NS/NR |
| 1. What is the highest level of education you are or have you attended?   ☐ Primary  ☐ EGB  ☐ Secondary  ☐ Polymodal  ☐ Tertiary  ☐ University  ☐ Postgraduate  ☐ Special Education  ☐ NS/NC (pase) |
| 1. Is that level over?   ☐ Yes  ☐ No  ☐ NS/NC |
| 1. What degree/orientation did you graduate from high school with?   ☐ Social sciences / humanities  ☐ Economics and administration (commercial expert)  ☐ Natural Sciences  ☐ Communication  ☐ Art  ☐ Physical Education  ☐ Foreign languages  ☐ Agricultural / Environment  ☐ Tourism  ☐ Computer Science  ☐ Technical Education (agricultural, industrial or services) |
| 1. What higher level degree (tertiary or university) did you receive from, and from which university/tertiary institute/teaching staff?   WRITE THE UNIVERSITY OR TERTIARY INSTITUTE / FACULTY WITH A PERIOD AFTER THE TITLE. |
| 1. *We would now like to refer to other organized learning activities in which you may have participated in the past 12 months, including work-related and non-work-related activities.*   During the last 12 months, have you participated in courses conducted through virtual education?  ☐ Yes  ☐ No  ☐ NS/NC |
| 1. During the past 12 months, have you attended any organized on-the-job training sessions or training conducted by supervisors or colleagues?   ☐ Yes  ☐ No  ☐ NS/NC |
| 1. During the last 12 months, have you participated in seminars or workshops on your own?   ☐ Yes  ☐ No  ☐ NS/NC |
| 1. During the past 12 months, have you participated in courses or private lessons that you have not yet reported?   ☐ Yes  ☐ No  ☐ NS/NC |
| 1. Was this activity mainly work-related?   ☐ Yes  ☐ No  ☐ NS/NC |
| 1. Could you specify more precisely the main reason for participating in this activity?   ☐ To do my job better and/or improve my career chances.  ☐ So I'm less likely to lose my job.  ☐ To increase my chances of getting a job, or changing jobs or professions.  ☐ To start my own business.  ☐ They forced me to participate.  ☐ To increase my knowledge or skills in a subject that interests me.  ☐ To obtain a certificate.  ☐ Other: |
| 1. This activity was carried out ...   ☐ Only during business hours  ☐ Mainly during working hours  ☐ Mainly outside working hours  ☐ Only after hours |
| 1. How useful was this training for the job or business you had at the time or still have? You would say that this was ...   ☐ Not at all useful  ☐ Something useful  ☐ Moderately useful  ☐ Very helpful |
| 1. Did your employer or prospective employer pay a fee or tuition, testing costs, book fees, or other costs associated with your participation in any of the above activities? That would be ...   ☐ Yes, totally  ☐ Yes, partially  ☐ No, not at all  ☐ There were no such costs  ☐ I didn't have an employer or potential employer at the time. |
| 1. **Job Surveyed** |
| *In this section you must answer for your MAIN JOB. If the respondent has two or more jobs simultaneously, record only the job that spends the most hours.*   1. What is the name of the occupation you do? |
| 1. What tasks do you perform in that job? |
| 1. What tools, equipment and/or machinery do you use? |
| 1. Do any of the tools, equipment, or machinery you use allow you to automate a task?   ☐ Yes  ☐ No  ☐ NS/NC |
| 1. What does the establishment in which you carry out your main work do or produce? |
| 1. That job does....   ☐ As an employer for your own business/company/activity  ☐ As a self-employed person for your own business/company/activity  ☐ For the business, company, activity of a family member  ☐ As a worker or employee for an employer/company/institution  ☐ As a domestic servant  ☐ NS/NC |
| 1. Including you, how many people work in that business, company, activity?   ☐ 1 alone (it is self-employed, without employees)  ☐ From 2 to 5  ☐ 6 to 10  ☐ 11 to 50  ☐ 51 to 200  ☐ More than 200  ☐ NS/NC |
| 1. Is the job of ...   ☐ Public Sector  ☐ Private sector  ☐ Worker Cooperative/ Mutual  ☐ Other non-profit organization (e.g., union, club, NGO)  ☐ Other [specify]  ☐ NS/NC |
| 1. Is this job ...   ☐ Permanent  ☐ With an end date (term contract) / temporary  ☐ Work or service lease  ☐ Changas Type  ☐ Employment Plan  ☐ Scholarship-Internship  ☐ NS/NC |
| 1. In your work... Do you supervise the work of others or tell them what to do? How many people do you supervise?   ☐ From 1 to 9 employees  ☐ More than 10 employees  ☐ Does not supervise  ☐ NS/NC |
| 1. In that work...   ☐ They give you discounts for retirement  ☐ You pay a single tax or self-employed  ☐ He does not have a pension contribution  ☐ NS/NC |
| 1. How many hours did you work last week at your main job? |
| 1. Last week, did you want to work more hours?   ☐ Yes  ☐ No  ☐ NS/NC |
| 1. All things considered, how satisfied are you with your current job? You would say that you are ...   ☐ Extremely satisfied  ☐ Satisfied  ☐ Neither satisfied nor dissatisfied  ☐ Dissatisfied  ☐ Extremely dissatisfied |
| 1. What was the amount of income/salary you received last month for your main occupation? We ask you to inform us of the net income, that is, that which you receive "in your pocket". |
| 1. What was the amount of income/salary you received last month for your main occupation? We ask you to inform us of the net income, that is, that which you receive "in your pocket". (For those who did not answer the previous question)   ☐ He had no income  ☐ 1 to 90,000  ☐ 90,001 to 150,000  ☐ 150,001 to 200,000  ☐ 200,001 to 270,000  ☐ 270,001 to 330,000  ☐ 330,001 to 410,000  ☐ 410,001 to 510,000  ☐ 510,001 to 660,000  ☐ 660,001 to 900,000  ☐ 900,001 to 1,400,000  ☐ 1,400,001 to 2,500,000  ☐ 2,500,001 to 5,300,000  ☐ More than 5,300,000  ☐ Ns/Nc |
| 1. What was the amount of household income your household received last month? (Consider both labor and non-labor income, adding up the income of all members who live with you in your household) |
| 1. What was the amount of family income you received last month? (Consider both labor and non-labor income, adding up the income of all the members who live with you in your household.) (For those who did not answer the previous question)   ☐ He had no income  ☐ 1 to 200,000  ☐ 200,001 to 400,000  ☐ 400,001 to 600,000  ☐ 600,001 to 800,000  ☐ 800,001 to 1,000,000  ☐ 1,000,001 to 1,200,000  ☐ 1,200,001 to 1,400,000  ☐ 1,400,001 to 1,600,000  ☐ 1,800,001 to 2,000,000  ☐ 2,000,001 to 2,200,000  ☐ 2,200,001 to 2,400,000  ☐ 2,400,001 to 5,000,000  ☐ More than 5,000,000  ☐ Ns/Nc |
| 1. **Tasks and activities at work (interaction, learning, organization, physical work)** |
| *The following questions refer to the various activities and tasks that you perform in your current job.*   1. To what extent can you choose or change the sequence of your tasks?   ☐ Not at all  ☐ Very little  ☐ To some extent  ☐ Largely  ☐ To a very high extent |
| 1. To what extent can you choose or change the way you do your work?   ☐ Not at all  ☐ Very little  ☐ To some extent  ☐ Largely  ☐ To a very high extent |
| 1. To what extent can you choose or change the speed or pace at which you work?   ☐ Not at all  ☐ Very little  ☐ To some extent  ☐ Largely  ☐ To a very high extent |
| 1. To what extent can you choose or change your working hours?   ☐ Not at all  ☐ Very little  ☐ To some extent  ☐ Largely  ☐ To a very high extent |
| 1. In your own work, how often do you learn new things related to your job from colleagues or supervisors?   ☐ Never  ☐ Less than once a month  ☐ Less than once a week but at least once a month  ☐ At least once a week but not every day  ☐ Every day |
| 1. How often does your job involve "learning by doing" the tasks you do?   ☐ Never  ☐ Less than once a month  ☐ Less than once a week but at least once a month  ☐ At least once a week but not every day  ☐ Every day |
| 1. How often does your job involve keeping up with new products or services?   ☐ Never  ☐ Less than once a month  ☐ Less than once a week but at least once a month  ☐ At least once a week but not every day  ☐ Every day |
| 1. What proportion of your time spent in the last week cooperating or collaborating with your coworkers?   ☐ No time  ☐ Up to a quarter of the time  ☐ Up to half the time  ☐ All the time  ☐ No Answer / No Answer |
| 1. How often do you share work information with your colleagues? *(if the interviewee has no colleagues, the answer should be "Never")*   ☐ Never  ☐ Less than once a month  ☐ Less than once a week but at least once a month  ☐ At least once a week, but not every day  ☐ Every day  ☐ No Answers/ No Answers |
| 1. How often in your work do you train or teach people, either individually or in groups?   ☐ Never  ☐ Less than once a month  ☐ Less than once a week but at least once a month  ☐ At least once a week, but not every day  ☐ Every day  ☐ No Answers/ No Answers |
| 1. How often in your work do you advise or advise people?   ☐ Never  ☐ Less than once a month  ☐ Less than once a week but at least once a month  ☐ At least once a week, but not every day  ☐ Every day  ☐ No Answers/ No Answers |
| 1. How often in your work do you plan your own activities?   ☐ Never  ☐ Less than once a month  ☐ Less than once a week but at least once a month  ☐ At least once a week, but not every day  ☐ Every day  ☐ No Answers/ No Answers |
| 1. How often in your work do you plan the activities of others?   ☐ Never  ☐ Less than once a month  ☐ Less than once a week but at least once a month  ☐ At least once a week, but not every day  ☐ Every day  ☐ No Answers/ No Answers |
| 1. How often in your work do you convince or influence other people?   ☐ Never  ☐ Less than once a month  ☐ Less than once a week but at least once a month  ☐ At least once a week, but not every day  ☐ Every day  ☐ No Answers/ No Answers |
| 1. How often in your work do you negotiate with people, whether inside or outside your company or organization?   ☐ Never  ☐ Less than once a month  ☐ Less than once a week but at least once a month  ☐ At least once a week, but not every day  ☐ Every day  ☐ No Answers/ No Answers |
| 1. How often does your job involve performing physical tasks for a long time?   ☐ Never  ☐ Less than once a month  ☐ Less than once a week but at least once a month  ☐ At least once a week, but not every day  ☐ Every day  ☐ No Answers/ No Answers |
| 1. How often does your job involve carrying or moving heavy loads?   ☐ Never  ☐ Less than once a month  ☐ Less than once a week but at least once a month  ☐ At least once a week, but not every day  ☐ Every day  ☐ No Answers/ No Answers |
| 1. How often does your current job involve being skillful or precise with your hands or fingers?   ☐ Never  ☐ Less than once a month  ☐ Less than once a week but at least once a month  ☐ At least once a week, but not every day  ☐ Every day  ☐ No Answers/ No Answers |
| 1. **Cognitive skills (reading, writing, numbers, problems)** |
| *The following questions pertain to the reading activities you do as part of your current job. Only report readings that are part of your current job, not readings you take outside of business hours. Also include any readings you take on computer screens or other electronic devices.*   1. In your current job, how often do you read commands or instructions?   ☐ Never  ☐ Less than once a month  ☐ Less than once a week but at least once a month  ☐ At least once a week, but not every day  ☐ Every day  ☐ No Answers/ No Answers |
| 1. In your current job, how often do you read letters, memos, or emails?   ☐ Never  ☐ Less than once a month  ☐ Less than once a week but at least once a month  ☐ At least once a week, but not every day  ☐ Every day  ☐ No Answers/ No Answers |
| 1. In your current job, how often do you read articles in newspapers, professional magazines, or brochures about your job?   ☐ Never  ☐ Less than once a month  ☐ Less than once a week but at least once a month  ☐ At least once a week, but not every day  ☐ Every day  ☐ No Answers/ No Answers |
| 1. In your current job, how often do you read manuals or reference material?   ☐ Never  ☐ Less than once a month  ☐ Less than once a week but at least once a month  ☐ At least once a week, but not every day  ☐ Every day  ☐ No Answers/ No Answers |
| 1. In your current job, how often do you read accounts, invoices, bank statements, or other financial statements?   ☐ Never  ☐ Less than once a month  ☐ Less than once a week but at least once a month  ☐ At least once a week, but not every day  ☐ Every day  ☐ No Answers/ No Answers |
| 1. In your current job, how often do you read diagrams, maps, or diagrams?   ☐ Never  ☐ Less than once a month  ☐ Less than once a week but at least once a month  ☐ At least once a week, but not every day  ☐ Every day  ☐ No Answers/ No Answers |
| *The following questions pertain to the writing activities you do as part of your current job. Include any writing activity you do on a computer screen or other electronic devices.*   1. In your current job, how often do you write letters, memos, or emails?   ☐ Never  ☐ Less than once a month  ☐ Less than once a week but at least once a month  ☐ At least once a week, but not every day  ☐ Every day  ☐ No Answers/ No Answers |
| 1. In your current job, how often do you write articles for newspapers, magazines, or newsletters?   ☐ Never  ☐ Less than once a month  ☐ Less than once a week but at least once a month  ☐ At least once a week, but not every day  ☐ Every day  ☐ No Answers/ No Answers |
| 1. In your current job, how often do you write reports?   ☐ Never  ☐ Less than once a month  ☐ Less than once a week but at least once a month  ☐ At least once a week, but not every day  ☐ Every day  ☐ No Answers/ No Answers |
| *The following questions refer to activities that you perform as part of your current job and that relate to numbers, quantities, numerical information, statistics, or mathematics.*   1. In your current job, how often do you calculate prices, costs, or budgets?   ☐ Never  ☐ Less than once a month  ☐ Less than once a week but at least once a month  ☐ At least once a week, but not every day  ☐ Every day  ☐ No Answers/ No Answers |
| 1. In your current job, how often do you use or calculate fractions, decimals, or percentages?   ☐ Never  ☐ Less than once a month  ☐ Less than once a week but at least once a month  ☐ At least once a week, but not every day  ☐ Every day  ☐ No Answers/ No Answers |
| 1. In your current work, how often do you use algebraic calculations or simple formulas?   ☐ Never  ☐ Less than once a month  ☐ Less than once a week but at least once a month  ☐ At least once a week, but not every day  ☐ Every day  ☐ No Answers/ No Answers |
| 1. In your current work, how often do you use more advanced mathematics or statistics, such as complex algebra, trigonometry, or regression techniques?   ☐ Never  ☐ Less than once a month  ☐ Less than once a week but at least once a month  ☐ At least once a week, but not every day  ☐ Every day  ☐ No Answers/ No Answers |
| *The following questions refer to the "problem-solving" tasks you perform in your current job. Think of "problem-solving" as what happens when you are faced with a new or difficult situation that requires you to think a little about what you will do next.*   1. How often, in your work, do you face relatively simple problems for which it takes you no more than 5 minutes to find a good solution?   ☐ Never  ☐ Less than once a month  ☐ Less than once a week but at least once a month  ☐ At least once a week, but not every day  ☐ Every day  ☐ No Answers/ No Answers |
| 1. How often, in your work, do you face more complex problems for which it takes you at least 30 minutes to find a good solution? The 30 minutes only refers to the time needed to THINK about a solution, not the time needed to implement it   ☐ Never  ☐ Less than once a month  ☐ Less than once a week but at least once a month  ☐ At least once a week, but not every day  ☐ Every day  ☐ No Answers/ No Answers |
| **Technology** |
| *The following questions pertain to using computers or the Internet as part of your current job. They do not refer to the use of computers or the Internet in any other work that you have had before your current job, or the use you may give it in other areas such as the home.*   1. Do you conduct transactions online, such as buying or selling products or services, or banking?   ☐ Never  ☐ Less than once a month  ☐ Less than once a week but at least once a month  ☐ At least once a week, but not every day  ☐ Every day  ☐ No Answers/ No Answers |
| 1. In your current job, how often do you use a spreadsheet program, for example, Excel?   ☐ Never  ☐ Less than once a month  ☐ Less than once a week but at least once a month  ☐ At least once a week, but not every day  ☐ Every day  ☐ No Answers/ No Answers |
| 1. In your current job, how often do you use a word processor, for example, Word?   ☐ Never  ☐ Less than once a month  ☐ Less than once a week but at least once a month  ☐ At least once a week, but not every day  ☐ Every day  ☐ No Answers/ No Answers |
| 1. In your current job, do you use a programming language to program?   ☐ Never  ☐ Less than once a month  ☐ Less than once a week but at least once a month  ☐ At least once a week, but not every day  ☐ Every day  ☐ No Answers/ No Answers |
| 1. In your current job, do you participate in real-time discussions over the Internet, e.g., online conferences, or chat groups?   ☐ Never  ☐ Less than once a month  ☐ Less than once a week but at least once a month  ☐ At least once a week, but not every day  ☐ Every day  ☐ No Answers/ No Answers |
| 1. What level of computer use is necessary to perform your current job?   ☐ BASIC, for example, using a computer for normal routine tasks like entering data or sending and receiving emails.  ☐ MODERATE, e.g., word processing, spreadsheets, or managing databases.  ☐ COMPLEX, for example, developing software, programming using language such as java, sql, php, perl, python or maintaining a complex computer network.  ☐ No Answers/ No Answers |
| **Use of artificial intelligence** |
| *Currently, the use of artificial intelligence (AI) has diversified, which makes it easier for us to create content, such as text, images, music, audio, and videos. The most popular application with this type of AI is ChatGPT, but there are other well-known applications such as Google BARD, Copilot, DALL-E, Midjourney, DeepL, etc. We are interested in knowing about the use you make of artificial intelligence in your work.*   1. Do you use any kind of artificial intelligence application in your work tasks?   ☐ Yes  ☐ No  ☐ NS/NC |
| 1. What are the main uses you give it? (multiple)   ☐ Writing / editing / correcting texts  ☐ Comprehension/synthesis of texts  ☐ Translating texts  ☐ Image recognition  ☐ Image and video generation/design  ☐ Audio Recognition / Audio to Text Conversion  ☐ Consult specific questions (chatbot)  ☐ Computer Code Programming  ☐ For performing mathematical calculations  ☐ Other uses |
| 1. What tools do you use?   ☐ ChatGPT  ☐ BARD  ☐ Microsoft Copilot  ☐ Github Copilot  ☐ DALL-E  ☐ Midjourney  ☐ DeepL  ☐ Civitai  ☐ Quillbot  ☐ Character.IA  ☐ Other: |
| 1. Considering the app you use the most in your work, how often do you use that app?   ☐ Never  ☐ Once a week or less  ☐ Several times a week  ☐ Every day  ☐ NS/NC |
| 1. Do you think incorporating AI into your daily tasks improved your work?   ☐ Improved considerably  ☐ Improved  ☐ It remained the same as before  ☐ It got worse  ☐ It got considerably worse  ☐ NS/NC |
| 1. Do you review the results you get from using AI in your work?   ☐ Always  ☐ Frequently  ☐ Sometimes  ☐ Rarely  ☐ Never  ☐ NS/NC |
| 1. Would you like to incorporate some form of AI into your current job? (for those who don't use AI)   ☐ Yes  ☐ No  ☐ NS/NC |
